# Interventions for Subjects with Depressive Symptoms with or without Unhealthy Alcohol Use: Are There Different Patterns of Change?

**DOI:** 10.3389/fpsyg.2017.00788

**Published:** 2017-05-23

**Authors:** Cecilie Skule, Pål Ulleberg, Torkil Berge, Hilde Dallavara Lending, Jens Egeland, Nils Inge Landrø

**Affiliations:** ^1^Department of Psychiatry, Diakonhjemmet HospitalOslo, Norway; ^2^Department of Psychology, University of OsloOslo, Norway; ^3^Psychiatric Department, Hospital of VestfoldTønsberg, Norway; ^4^Clinical Neuroscience Research Group, Department of Psychology, University of OsloOslo, Norway

**Keywords:** depression, alcohol use, control of depression, cognitive therapy

## Abstract

**Background:** It has been suggested that alcohol problems negatively affect therapeutic interventions for depression. This study examines the patterns of change in depressive symptoms following an intervention for depression, in participants with or without comorbid unhealthy alcohol use.

**Methods:** Depressive symptoms (BDI–II), perceived control of depressive symptoms (UNCONTROL) and unhealthy alcohol use (AUDIT) were assessed in 116 patients before and after attending a cognitive behavioral psychoeducational intervention for depression. At pretest the mean score of AUDIT was 8.1, indicating a, on average, risk of harmful level of alcohol abuse. At pretest the majority of the total sample had a moderate degree of depressive symptoms, with a mean BDI–II score of 25.1 and 36.2% had a risky use of alcohol as measured with AUDIT score at 8 points or above. To assess the relationship between depressive symptoms, perceived uncontrollability of depression and alcohol use across time, a cross-lagged panel model was estimated.

**Results:** A clinical significant reduction of depressive symptoms, and a parallel and statistically significant increase in the perceived control of depressive symptoms, was identified after attending a cognitive behavioral psychoeducational intervention for depression. At posttest, the mean BDI–II score was 17.8, demonstrating a statistically significant decrease of 7.3 points in depressive symptoms from before starting the course to 6 months later. The effect size (*d*-value) of 0.83 can be interpreted as a large decrease in depressive symptoms. In this sample alcohol use and depressive symptoms seemed to be unrelated. The cross-lagged correlation panel analysis indicated that a high degree of perceived control of depressive symptoms leads to a reduction in depressive symptoms, and not vice versa.

**Conclusion:** We found that this intervention for depression were effective in reducing depressive symptoms. The patterns of change seemed to be independent of risky use of alcohol, although leaving the study was systematically associated with higher AUDIT-scores. As participants with or without unhealthy alcohol use show the same patterns of change regarding reduction of depressive symptoms and perceived control of depression, both groups could be offered the same cognitive behavioral psychoeducational interventions for depression.

## Introduction

Depression is a highly prevalent and recurrent disorder, often comorbid with alcohol-related problems (Ostacher, 2007; Palfai et al., 2007; Seignourel et al., 2008). Conversely, a majority of patients seeking treatment for alcohol problems show clinically significant levels of depressive symptoms (Brown et al., 1997). In a meta-analysis of treatment of comorbid alcohol use disorders and depression, the authors refer to a prevalence of up to 50% for depression in clinical populations with alcohol problems, and a lifetime risk of alcohol problems in clinical population with depression up to 40% (Riper et al., 2014). Prevalence of depression in adult population in Oslo during a 12 months' period is estimated to 7% (Kringlen et al., 2001). Theories of causality in dual diagnosis do share the intention of finding the best way to understand and treat patients with comorbid mental illness and alcohol or substance abuse disorders. Kushner and Mueser (1993) describe four different theoretical models aiming to explain the theoretical relationship between mental disorders and substance abuse disorders.

One model is based on the assumption that substance abuse has developed as an attempt to relieve symptoms related to the primary mental problem, an example being the self-medication hypothesis (Kanthzian, 1997). However, this hypothesis has not been supported in systematic studies (Lagoni et al., 2011). Another theoretical model is based on the premise that mental disorder is a secondary consequence of substance abuse. This model has also been difficult to support (Seignourel et al., 2008). A third theoretical model is the common factor model, explaining the high comorbidity of mental disorders and substance abuse by different underlying factors of either genetic, neurobiological, psychological, or social origin. Findings supporting a common factor model are not consistent, but there seem to be some empirical support for both the neurobiological or hypersensitivity model, and further a higher risk of both mental disorders and substance abuse problems in patients diagnosed with anti-social personality disorder (Kushner and Mueser, 1993; Thylstrup et al., 2015). The fourth theoretical model is less oriented to temporal and direct causality, emphasizing the sustaining factors in the interaction of the comorbidity (Kushner and Mueser, 1993). Recently, this interactionist perspective is referred to as the most theoretically and clinically meaningful model (Mathias et al., 2008; Medhus, 2014; Morisano et al., 2014). By including different causal mechanisms in the complicated neurobiological, somatic, psychological and social interplay between the mental disorders and substance abuse disorder, this model considers the variability and mutuality of the two disorders.

There is not a clear consensus on how alcohol problems influence the course and treatment outcome of depression, and how symptoms of depression impact the course and treatment of alcohol problems. It has been suggested that the interactive nature of the two disorders leads to poorer outcomes of treatment for both depression and substance abuse (Grella and Stein, 2006; Boden and Moos, 2009). Some authors argue that even alcohol use at a subclinical level may impede the treatment of depression (Ramsey et al., 2005). Comorbid alcohol problems have in several studies been associated with poorer course of treatment for depression (Sullivan et al., 2005). On the other hand, a meta-analysis demonstrated a reduction both in depressive symptoms and substance abuse with treatment with anti-depressants for patients with alcohol or drug dependence, and the authors claimed that alcohol abuse or other forms of substance abuse should not be a barrier to treatment of depression (Nunes and Levin, 2004). In example, a study by Watkins et al. (2011) showed positive effects of a cognitive behavioral program for depression for patients in a residential substance abuse clinic. In a meta-analysis on treatment of comorbid alcohol problems and depression, Riper et al. (2014) found positive effects on depressive symptoms and unhealthy alcohol use by different psychotherapeutic interventions, but did not identify integrated treatment as superior to a single focused treatment.

Psychoeducational interventions that strengthen patients' awareness of associations between negative patterns of feeling, thinking and behavior—in example the connection between dysphoric mood, depressive rumination, reduced level of activity, and diminished problem solving capacity—can be helpful for patients with depression (Cuijpers, 1998; Swan et al., 2004; Watkins et al., 2011; Riper et al., 2014). Cognitive behavioral therapy and psychoeducation may increase the awareness of early signs and symptoms of relapsing, and strengthen self-efficacy in coping with depression (Bockting et al., 2009; Tursi et al., 2013). It has been suggested that psychological treatments work through changes in perceived control of depressive symptoms. The perception of depressive symptoms as highly aversive and uncontrollable can lead to “depression about depression,” which can be reduced by increasing the perceived controllability of depressive symptoms (Teasdale, 1985, 1999; Teasdale et al., 2001).

There is strong documentation for the effectiveness of psychosocial treatments, such as cognitive behavioral therapy (Oei and Dingle, 2008; Cuijpers et al., 2013; Hans and Hiller, 2013) and psychoeducation for depression (Clark et al., 2008). However, research on psychosocial treatments for patients with comorbid depression and substance use disorder are limited (Clark et al., 2008). Some studies have reported positive effects of a cognitive behavioral program for depression for patients in a residential substance abuse clinic (Watkins et al., 2011) and positive effects of different psychotherapeutic interventions, such as cognitive behavioral therapy and motivational therapy (Riper et al., 2014). Studies of depressive symptoms and perceived control of depressive symptoms in a sample of patients with depressive symptoms with or without unhealthy alcohol use indicated similarities between these groups, rather than differences (Skule et al., 2014a,b). The intervention in this study contains elements that have been found helpful for patients with depression (Clark et al., 2008).

This study examines the patterns of change in depressive symptoms following an outpatient cognitive behavioral, psychoeducational intervention for depression, in participants with or without comorbid unhealthy alcohol use. With the presented documentation for the effects of psychoeducational interventions for depression in mind, and similarities in symptom profile and perceived control of depression, we would expect that the patterns of change are independent of comorbid alcohol problems.

## Materials and methods

### Participants

This is a multicenter study with a sample based on participants from treatment centers from the South Eastern Region of Norway and from the Students mental health service at the University of Oslo. The sample consisted of 116 patients seeking help for depressive symptoms in the mental health care system, including substance abuse clinics. The participants attended a cognitive behavioral psychoeducational intervention. The sample comprised 60.5% females and 39.5% males, who were aged between 19 and 63 years (*M* = 44.1, *SD* = 13.7). The majority of the sample was married or cohabited with a stable partner (42%), whereas 35% were single. A total of 56% of the sample had a university degree. The proportion of the sample with a comorbid unhealthy alcohol use was 36.2%. Based on their initial score on the AUDIT-measure of alcohol use, the sample was split in the three categories low risk (63.8%), medium risk (20.7%), and high risk of harmful alcohol use (15.5%). A Chi-square analysis showed that females were more represented in the low risk group compared to men, 70.8 and 29.2%, respectively (*p* < 0.001). Males were on the other hand more represented in the high-risk group compared to women; 55.6 and 44.4%, respectively. Otherwise, no statistically significant differences in age, marital status or education were found between the three AUDIT-categories.

Most of the patients were partly recruited from community mental health centers, and partly from substance abuse clinics. The intervention was offered participants that had experience with depressive symptoms. Exclusion criteria were psychotic symptoms or acute suicidal symptoms. See Table 1 for a description of patient characteristics.

**Table 1:** **Patient demographics, BDI–II-score, and UNCONTROL-score before treatment (*N* = 116)**.

Age (M, SD)	44.1 (13.7)
**GENDER**	
% Men	39.5
% Women	60.5
**HIGHEST EDUCATIONAL LEVEL ATTAINED**	
% Lower secondary	4.5
% Upper secondary/vocational	17.0
% Upper secondary/academic	22.3
% Tertiary	56.3
**MARITAL STATUS**	
% Single	34.9
% Married/reg. partner	41.6
% Divorced	20.4
% Widow/widower	3.5
BDI–II-score before (M, SD)	25.1 (11.3)
UNCONTROL-score before (M, SD)	40.9 (11.2)


### Attrition from pre- to post-test

**Figure 1:**
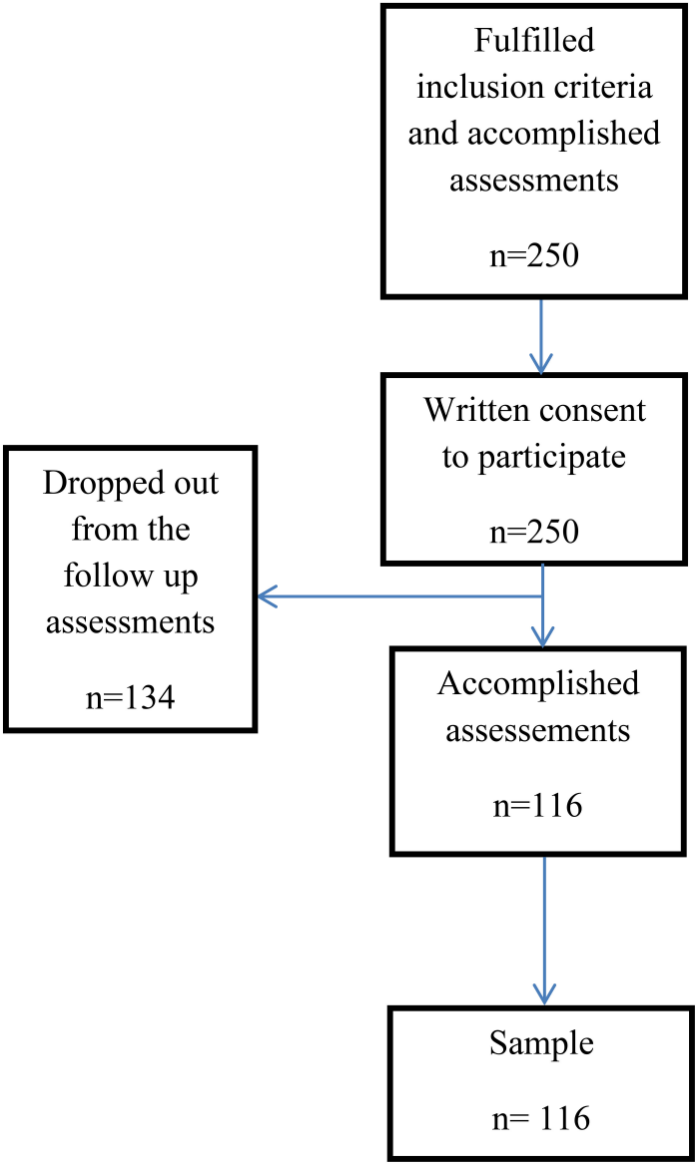
**Sample recruitment**.

Participants in this follow-up survey consisted of patients tested before the psychoeducational group started and 4 months after the psychoeducational group ended, a period of 6 months from pre- to post-testing, with an attrition rate of 53.6%, see Figure 1. As patients with substance use disorders are associated with poorer socioeconomic situation than other samples (Skule et al., 2014b), we controlled for such possible bias in the studied sample. The sample in the follow-up study did not differ statistically significantly from the pretest sample on patient characteristics or demographic variables like age, gender, level of education or marital status. Unhealthy use of alcohol, as measured by AUDIT-score was the only factor that was statistically significantly related to attrition from the research project.

### Instruments

#### The beck depression inventory—second edition (BDI–II)

Beck et al. (1996) is one of the most commonly used self-report instruments to estimate the severity of depression. The total score gives an indication of a mild, moderate or major depression. BDI–II consists of 21 items. Every item has four answering alternatives and is scored from 0 to 3. The maximum score is 63. The following values are recommended: total score 0–13 minimal depression, 14–19 mild, 20–28 moderate, and 29–63 refers to major depression. The reliability estimated by Cronbach's alpha was 0.914 before the course started, and 0.935 6 months after the course started.

#### Alcohol use disorders identification test (AUDIT)

Babor et al. (2001), has been developed by the World Health Organization, and consists of ten items. The AUDIT can be administered as an interview or self-administered by the patient. Each item is scored on a 5-point scale. The total score has a range from 0 to 40, where total scores of 8 or more are recommended as indicators of harmful alcohol use, as well as possible alcohol dependence. The following categories have been identified (Clark et al., 2008). Total score 0–7 low risk, 8–15 medium risk, 16–40 high risk.

#### Perceived uncontrollability of depression (UNCONTROL)

Teasdale (1985) consists of ten items that indicate the degree of perceived control of depressive symptoms, i.e., “When I'm depressed, there are things I can do to change how I feel,” “When I'm depressed, by changing the way I think I can change the way I feel” and “I feel hopeless about ever mastering depression.” Each item is scored on a 7-point scale. The total score has a range from 0 to 70, where higher scores reflect greater perceived controllability of depression. The reliability of the summated score, estimated by Cronbach's alpha, was 0.894 before the course started, and 0.910 6 months after the course started.

### Procedure

The project was approved by Regional Committees for Medical and Health Research Ethics no. 2009/1018. This study was carried out in accordance with the recommendations from the Regional Committees for Medical and Health Research Ethics with written consent from all subjects. All subjects gave written consent in accordance with the declaration of Helsinki. In addition to screening depressive symptoms, substance abuse and perceived uncontrollability of depression, the participants answered questions about demographic issues. Recruitment of participants in the research project took place in a clinical setting, at the time where the participants were informed about the course “Coping and relapse prevention of depression.” Most of the patients at the course were recruited by their therapists. All participants in the research project were asked to participate in the study by the group leaders as they enrolled the course, and they were informed that not attending the research study would have no consequences for their further treatment. Most of the participants were recruited directly from treatment centers and they were thus engaged in or had recently attended other forms of treatment. Information about patients who did not want to engage in the research project was not collected. The patients completed the screening before the cognitive behavioral intervention started.

### Intervention

A cognitive behavioral psychoeducational course, influenced by the “Coping with depression course” (Lewinsohn et al., 1984), consisted of eight meetings of 2 h each. Group leaders attended meetings were the manual were made known and discussed, but a systematically fidelity scale for implementation of the program was not developed. Before attending the intervention, individually or in groups, participants were informed about the program, the structure and length of the interventions, and the role of the group leaders. The group normally consisted of up to 12–15 participants, with some groups smaller, while some of the groups had up to 20 participants. The group leaders were two therapists; most often both were clinical psychologists, but some of the groups had other health professionals, i.e., a nurse or a doctor. Group leaders remained the same throughout the course. The group meetings were highly structured, with information about depressive symptoms, typical thinking patterns related to maintenance of depressive symptoms, loss of interest in activity, and family and social settings. Comorbid alcohol or substance abuse was addressed, but interventions directed toward changing alcohol patterns were not actively used during the sessions. Through the course, the cognitive behavioral model was presented, and the group leaders used it actively when participants were willing to share experiences in the groups. Relapse prevention was also a part of the program. The program is summarized in Table 2. For more information about the sample, instruments, and methods (Skule et al., 2014a,b, 2016).

**Table 2:** **Description of the intervention**.

**PROGRAM COGNITIVE BEHAVIORAL PSYCHOEDUCATIONAL INTERVENTION**
Characteristics of depression, prevalence and risk of relapse
Vulnerability of depression, triggering and sustaining factors
Depression and typical thought patterns. Rumination and worries
Suicidal thoughts and suicide attempts
Anxiety disorders and depression. Coping with anxiety
Activity as antidote against depression
Coping with sleep problems
Depression and the relation to family and friends
Antidepressant drugs. Cooperation with the doctor
Depression and substance abuse
Relapse prevention


### Statistical analysis

Preliminary analyses showed that the distribution of scores on BDI–II and UNCONTROL were in the acceptable range, with skewness values ranging from −0.03 to −0.51 and kurtosis values from 0.00 to −0.68. The AUDIT-scores did however have a non-normal distribution on both points in time (skew: 1.31 and 1.29, kurtosis 1.15 and 1.14). The AUDIT-scores were therefore log transformed to approximate a normal distribution (skew: −0.41 and −0.32, kurtosis: −0.43 and −0.55) before proceeding with further analyses.

**Figure 2:**
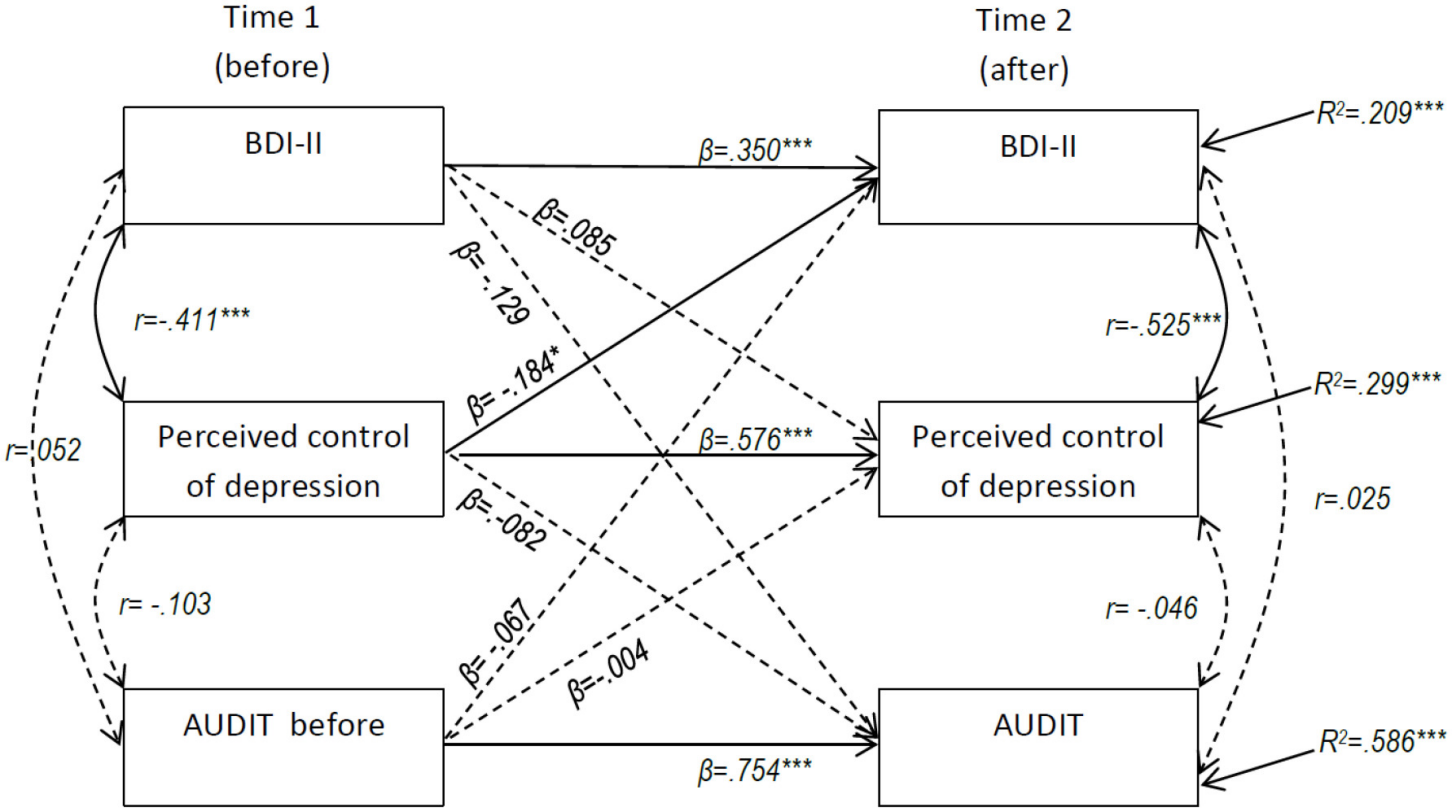
**Maximum likelihood estimation of cross lagged effects for change in BDI–II, perceived control of depression, and AUDIT over time (*N* = 116)**. Standardized coefficients reported. ^*^p < 0.05, ^***^p < 0.001.

To assess the relationship between depressive symptoms, perceived uncontrollability of depression and alcohol use across time, a cross-lagged panel model was estimated (Locascio, 1982). The model (presented in Figure 2) was tested by allowing depressive symptoms, perceived uncontrollability of depression and alcohol use at Time 1 (before the course started) to predict each other at Time 2 (6 months later). At the same time, the stability of depressive symptoms, perceived uncontrollability of depression and alcohol use across time was controlled for in the model. Furthermore, concurrent associations (i.e., correlations at the same point in time) between depressive symptoms, perceived uncontrollability of depression and alcohol use were estimated. This reciprocal model was thus estimated as a fully saturated model, i.e., a model with zero degrees of freedom are zero and will therefore have a perfect fit to the data. The model was estimated using the software Mplus 7.11 (Muthén and Muthén, 2010).

## Results

As presented in Table 3, alcohol use pattern as measured by AUDIT was not related to patients' scores on BDI–II or to their scores on UNCONTROL at either point in time. Dividing the participants into three AUDIT-categories (mild, moderate, and severe risky alcohol use) and performing a simple one-way ANOVA gave the same conclusion; no statistically significant differences in mean scores on BDI–II and UNCONTROL between the three AUDIT-categories were found at either point in time. Scores in BDI -II and UNCONTROL had a negative statistical significant correlation when measured at the same point in time, meaning that a high degree of depressive symptoms was related to a low degree of perceived control of depressive symptoms.

**Table 3:** **Zero-order correlations between BDI–II, AUDIT, and UNCONTROL measured before the course started to ~6 months later (*N* = 116)**.

	**BDI–II Pre**	**BDI–II Post**	**UNCONTROL Pre**	**UNCONTROL Post**	**AUDIT Pre**	**AUDIT Post**
BDI–II Pre	0.914					
BDI–II Post	0.422^*^	0.935				
UNCONTROL Pre	−0.411^*^	−0.321^*^	0.894			
UNCONTROL Post	−0.152	−0.540^*^	0.541^*^	0.910		
AUDIT Pre^a^	0.052	−0.030	−0.103	−0.059	0.918	
AUDIT Post^a^	−0.056	−0.037	−0.107	−0.094	0.756^*^	0.888
Mean	25.1	17.8	40.9	47.5	8.1	7.8
SD	11.3	12.0	11.2	10.8	(7.9)	(7.3)

*Cronbach's alpha values reported along the diagonal*.

* *p < 0.001*.

a *Logarithmic transformed AUDIT-scores used in the correlation analyses, mean, and standard deviation estimated on the basis of raw AUDIT-scores*.

The results presented in Table 4 demonstrated a statistically significant decrease of 7.3 points in depressive symptoms from before starting the course to 6 months later. The effect size (*d*-value) of 0.83 can be interpreted as a large decrease in depressive symptoms according to Cohen's criteria (Cohen, 1988). On average, the patients move from middle/upper part of the moderate level of depressive symptoms to mild level of depressive symptoms The distribution within the minimal, mild, moderate and major depression categories before the course started was 12.9, 18.1, 28.4, and 40.5%, respectively. Six months later, the corresponding percentages within these categories were 40.5 (minimal), 18.1 (mild), 21.6 (moderate), and 19.8 (major depression).

**Table 4:** **Change in mean scores on depressive symptoms (BDI–II), perceived uncontrollability of depression (UNCONTROL), and alcohol use (AUDIT)**.

	**Before**		**6 months later**		***t***	***d*-value**
	**Mean**	**(SD)**	**Mean**	**(SD)**		
BDI–II	25.1	(11.3)	17.8	(12.0)	6.20^*^	0.83
UNCONTROL	40.9	(11.2)	47.5	(10.8)	−0 6.72^*^	0.89
AUDIT	8.1	(7.9)	7.8	(7.3)	0.19^a^	0.06

*Paired samples t-test (N = 116)*.

* *p < 0.001*.

a *t-value estimated on the basis of logarithmic transformed AUDIT-scores*.

The patients also had a statistically significant increase in mean score on perceived controllability of depression, meaning that they on average perceived more control of depression after treatment. This increase can be described as large according to Cohen's criteria, *d* = 0.89. No statistically significant change in alcohol pattern measured through AUDIT was found. The AUDIT-score was relatively stable over the 6 month period, *r* = 0.81 (Table 2). Before the treatment was started, the distribution within the three AUDIT-categories low, medium, and high risk was 63.8, 20.7, and 15.5%, respectively. Six months later, only trivial changes in the corresponding percentages were observed: 64.7% (low risk), 21.6% (medium risk), and 13.8% (high risk).

The results from the estimated cross-lagged model are presented in Figure 2. All three measures showed moderate to high stability over the 6 month period. The significant negative cross-lagged relationship between perceived uncontrollability of depression at Time 1 and BDI–II score at Time 2 indicates that a high degree of perceived controllability of depression leads to a decrease in depressive symptoms 6 months later in time. The non-significant cross lagged relationship between BDI–II score at Time 1 and perceived controllability at Time 2 does not support the notion that the degree of depressive symptoms affects perceived controllability of depression at a later point in time. Risky alcohol use was not significantly related to change in either BDI–II or perceived controllability of depressive symptoms over time. Gender was also included as a covariate in the model in, but only showed a statistically significant relationship with alcohol use at Time 1, and was therefore excluded from the model.

## Discussion

The main finding was that the intervention influenced depressive symptoms significantly in subjects both with and without unhealthy alcohol use. This reduction was controlled by testing the effect of AUDIT, both categorical and dimensional. Further, the intervention influenced the degree of perceived control of depressive symptoms independently of alcohol use before treatment. The associations between BDI–II and UNCONTROL were negatively correlated, meaning that reduced depressive symptoms and increased belief in coping skills followed each other systematically. A high degree of perceived control of depression at pretest was related to a decrease of depressive symptoms 6 months later.

The mean reduction of depressive symptoms following the attendance of the course was ~7.3 points measured with BDI–II. At pretest the participants reported a significant burden of depressive symptoms compared to samples usually studied (Teasdale et al., 2001). The sample is characterized by long-lasting symptoms of depression. The therapeutic intervention in this study was directed to persons with experience of depressive symptoms. It is not likely that the participants attend this kind of an intervention at a time when their symptoms are at a maximum level. This makes the possible effect of “regression toward the mean” a less plausible explanation of the reduced symptom load of depressive symptoms.

Attending the intervention did not influence the AUDIT-scores in patients with or without unhealthy alcohol use, during the 6 months' period of this study. The decrease in depressive symptoms occurred in the context of no decrease in alcohol use. The lack of change in alcohol pattern can be related to the relative big sample of participants with low risk. On the other hand, not even the part of the sample with moderate to high risk of problematic drinking had a change in their alcohol use pattern. Riper et al. (2014) introduces the possibility of a “sleeper effect” as an explanation of a delayed effect of cognitive behavioral treatment of alcohol problems. They found that the effect on depressive symptoms appeared earlier than the effects on the alcohol problems, and claimed that the effect of cognitive therapy might strengthen over time. Such an explanation might be relevant for the interpretation of the results in our study as well, but was not examined in this sample.

The relief from depressive symptoms and the improved perceived control of depressive symptoms are in line with findings in other studies of the effect of cognitive behavioral therapy (Barber and DeRubeis, 1989; Cuijpers, 1998; Clark et al., 2008; Tursi et al., 2013). It is assumed that psychological treatments operate through relatively specific therapeutic processes, but at the same time apparently diverse treatments often have similar effects. Teasdale (1985, 1999) describes a depression-cognition vicious circle—“depression about depression”—where depressive symptoms are maintained because they are experienced as uncontrollable. Different kinds of treatments may have an effect by providing skills that reduce symptoms of depression, thus strengthening a sense of efficacy in controlling depression. A variety of skills can have this effect, such as constructive or pleasant activity, exercise, positive social interaction, modifications of thought content, reducing depressive rumination or changing patients' relationship to their dysfunctional thoughts and feeling.

Perceived illness control has also been connected to prevention of depressive symptoms and better adjustment in chronic illnesses, i.e., rheumatoid arthritis (Chaney et al., 1996). These diseases do often have an unpredictable and intermittent nature, and an important goal is to enhance patients' perceptions of personal control over daily aspects of disease management. Likewise, attributions of uncontrollability for negative situations increase the risk to develop depressive symptoms in people with a negative explanatory style, causing a feeling of helplessness and hopelessness (Abramson et al., 1978; Sanjuán and Magallares, 2009). Conversely, a preventive mechanism to relapse or recurrence of depression may be the ability to notice, but not overreact to, negative bodily sensations and experiences (Beshai et al., 2011). A belief in the possibility to control the depressive symptoms could be an important part of this ability. Control over depression may also be related to the concept of self-efficacy, defined as a belief in one's ability to succeed in specific situations or accomplish a task, and to view difficult tasks as something to be mastered rather than something to be avoided (Bandura, 1977). Participants with high self-efficacy and a belief in that it is possible to control depressive symptoms, may be more likely to make good use of the cognitive behavioral psychoeducational intervention for depression.

In contrast to other studies (Brown et al., 1997; Ramsey et al., 2005) the results from this study did not identify unhealthy alcohol use as a barrier to positive treatment outcome for depression. On the other hand, the attrition rate was considerable, and systematically related to increased AUDIT—score. The selection of the sample toward fewer participants with high AUDIT—score, further restricts generalization of the results. Still, a part of the sample in the follow up study did have an AUDIT—score that indicated alcohol dependence. Further objections toward this conclusion may be that the sample consisted of participants with a wide variety of possible harmful alcohol use.

Sullivan, Fiellein and O'Connor (Sullivan et al., 2005) claim that most of the research until 2005 has excluded patients with less severe alcohol problems and a primary care outpatient setting. To our knowledge, there are no previous studies following a sample consisting of depressive subjects with and without unhealthy alcohol use participating in an intervention aiming toward reduced depressive symptoms and increased perceived control of symptoms.

The results of this study indicate that alcohol pattern is not influenced by severity of depressive symptoms, and do not strengthen or weaken the possibility of alcohol problems leading to depressive symptoms. The findings cannot exclude any associations between depressive symptoms and alcohol use described in the four theoretical models of Kushner and Mueser (1993).

### Clinical implications

The results provide the basis for challenging routines where patients with depressive symptoms are given different and often separate treatments, depending on their pattern of alcohol or substance use. This clinical practice can have unintended negative effects, such as excluding subjects with unhealthy alcohol use who can benefit from therapeutic interventions if they are included. Further, the findings emphasize the need for a direct intervention toward the alcohol problems. Treating depressive symptoms do not in itself solve problems with unhealthy alcohol use.

### Limitations

The participants in this study did not undergo a formal diagnostic interview, but severity of depressive symptoms (BDI–II) and pattern of unhealthy alcohol use (AUDIT) were examined by well-known instruments with high validity and reliability. The main aim of this study was to investigate possible differences between participants with or without unhealthy alcohol use relating patterns of change in depression severity after attending the programme “Coping and relapse prevention of depression.” Comparison of the effect of this intervention with other therapeutic interventions for depressive symptoms was not a target for this study.

Another limitation is the lack of a strict implementation plan, for instance assessments for treatment implementation. We developed a detailed manual developed for the intervention, but we could not exclude that possibility of systematic differences between the treatment sites.

Attrition rate from pre- to post-test was substantial, 53.6%, and statistically significant related to AUDIT-score, indicating that increasing severity of alcohol problems were clearly related to drop out from the research project. The statistically significant relation between increased severity of alcohol problems and increased attrition rate suggest that participants with serious alcohol problems had problems with completing the study. The sample in the follow up study became a selected sample since severity of alcohol pattern was related to the dropout rate. This is an obvious limitation when it comes to the interpretation of the findings, and leaves in doubt the pattern of change in participants with severe alcohol problems. Although, follow-up analyses among the participants with the most severe level of alcohol problems (i.e., AUDIT-score of 16 or higher) showed the same decrease in depressive symptoms as the other participants, we cannot exclude the possibility that participants with high AUDIT—scores were systematically different from those who dropped out, for instance having a different motive for using alcohol. Thus, future studies should be aimed at investigating changes in depressive symptoms in patients with severe alcohol problems.

AUDIT is a screening instrument that aims to catch drinking pattern the last 12 months. The 12 months' instruction in the AUDIT-manual gives substantial limitations at posttest, as the time between pretest and posttest is only 6 months in this study. Despite this methodological weakness, we assume that participants' response to AUDIT could indicate their ongoing alcohol pattern and the changes in alcohol pattern, between pre- and posttest. Although our study has the limitation described, other studies of change in alcohol pattern over time in patients with comorbid mental disorders, have used AUDIT at 3 and 6 months follow up (Watkins et al., 2006; Wusthoff et al., 2014). However, this is a limitation that must be taken into consideration when interpreting the changes in BDI–II and UNCONTROL associated with attending the psychoeducational group program, as we did not have a full overview of the alcohol pattern during this period. The time of follow up of the participants did not allow us to detect a possible effect on the alcohol use that might have evolved later in a process of change, as described by Riper et al. (2014).

Another possible limitation regarding the use of AUDIT was the lack of information regarding motives for alcohol use. In a sample of nondependent alcohol users, differences in coping motives were not related to the amount of alcohol intake (Thomas et al., 2014). Some of the subjects in our sample with AUDIT-scores of 8 point and above might have a drinking pattern resembling the drinking pattern in the general population.

Limitation in the study design restricts the possibility of developing support for integrated treatment for depression and alcohol problems in favor of treatment addressing only depressive symptoms.

## Conclusion

In a sample were alcohol use and depressive symptoms seemed to be unrelated, we found that cognitive behavioral psychoeducational interventions for depression were effective in reducing depressive symptoms. In the sample that participated both in the pre- and posttest, the patterns of change seemed to be independent of risky use of alcohol. The intervention is related to the same degree of reduction in depressive symptoms and strengthens their perceived control of depressive symptoms in the same way as patients without this comorbidity. A high level of perceived controllability of depression at pretest is related to reduction of severity of depressive symptoms at posttest. The findings indicate that belief in coping capabilities will affect the efficacy of therapeutic interventions. Treatment addressing depressive symptoms was not sufficient to solve problems with alcohol use in patients with depressive symptoms and alcohol problems during the period we followed the sample.

## Author contributions

JE was central in the design of the study. CS and TB conducted literature searches and provided summaries of previous research studies. PU conducted the statistical analysis. HL participated in statistical analysis and the interpretation of the analysis CS wrote the first draft of the manuscript and all authors contributed to and have approved the final manuscript. NL contributed to the manuscript and supervised the writing process.

## Funding

The research was funded by the Regional Competence Centre for Dual Diagnoses, South Eastern Norway and the research fund in the Community Mental Health Center, Vinderen, Diakonhjemmet Hospital, Oslo. The funders had no role in study design, data collection, and analysis, decision to publish, or preparation of the manuscript.

### Conflict of interest statement

The authors declare that the research was conducted in the absence of any commercial or financial relationships that could be construed as a potential conflict of interest.
